# Anxiety, Depression, and Expanded Disability Status Scale Independently Predict the Perception of Disability in Persons With Multiple Sclerosis: A Cross-Sectional Study

**DOI:** 10.1155/bn/2744955

**Published:** 2025-01-10

**Authors:** Chiara Curatoli, Alessia Marcassoli, Filippo Barbadoro, Arianna Fornari, Matilde Leonardi, Alberto Raggi, Silvia Schiavolin, Rachele Terragni, Carlo Antozzi, Laura Brambilla, Valentina Torri Clerici, Paolo Confalonieri, Renato Mantegazza, Martina Lanza

**Affiliations:** ^1^Department of Neurosurgery, Fondazione IRCCS Istituto Neurologico Carlo Besta, Milan, Italy; ^2^Neurology, Public Health and Disability Unit, Fondazione IRCCS Istituto Neurologico Carlo Besta, Milan, Italy; ^3^Neuroimmunology and Neuromuscular Diseases Unit, Fondazione IRCCS Istituto Neurologico Carlo Besta, Milan, Italy

**Keywords:** biopsychosocial model, cognitive functioning, disability, multiple sclerosis, World Health Organization Disability Assessment Schedule 2.0

## Abstract

Multiple sclerosis (MS) is the most common cause of disability in young adults due to several motor, sensory, and cognitive symptoms. However, little is still known about the impact of psychological, cognitive, and social-support variables on subjective disability. This study is aimed at exploring the role of clinical, psychological, cognitive, and social-support variables in predicting disability levels as perceived by persons with multiple sclerosis (pwMS). The World Health Organization Disability Assessment Schedule (WHODAS 2.0) and the Expanded Disability Status Scale (EDSS) were used as subjective and objective measures of disability, respectively. State-Trait Anxiety Inventory and Beck Depression Inventory-II assessed symptoms of anxiety and depression; 19-item Medical Outcome Study–Social Support Survey assessed social support; and Rao's Brief Repeatable Battery assessed cognitive functioning. A multivariable regression analysis was applied using the WHODAS 2.0 as an outcome. One hundred and fifty-one pwMS (93 females, mean age 51.6, standard deviation (SD) 5.8) were enrolled. EDSS (*β* = 7.190; *p* < 0.001), state anxiety (*β* = 0.265; *p* = 0.009), and symptoms of depression (*β* = 0.835; *p* < 0.001) explained a large amount of the variance of subjective disability (Adj.*R*^2^ = 0.705; *p* < 0.001) measured through the WHODAS 2.0. Contrarily, cognitive functioning and perceived social support are not independently associated with the WHODAS 2.0 score. Psychosocial interventions in rehabilitation settings, aimed at reducing the overall perceived disability of pwMS, should be implemented in rehabilitation programs.

## 1. Introduction

Multiple sclerosis (MS) is a chronic autoimmune disease of the central nervous system, characterized by inflammation, demyelination, and axonal neurodegeneration. Its typical onset occurs in young adulthood, between 20 and 40 years, with the highest prevalence in women [1]. MS clinical manifestation is heterogeneous, involving a wide range of symptoms, mostly connected to the general chronic inflammation and the lesion site. Therefore, the clinical profile of persons with multiple sclerosis (pwMS) is characterized by motor (i.e., spasticity and muscle weakness), cerebellar (i.e., vertigo and ataxia), sensory (i.e., loss of sensation and paresthesia), visual (i.e., optic neuritis), brainstem (i.e., diplopia, trigeminal neuralgia, and dysphagia), and sphincter (i.e., bladder and bowel dysfunction) impairments [2]. Furthermore, 40%–70% of pwMS also present deteriorations in cognitive functioning, varying according to MS type and course [3]. Specifically, the most affected cognitive domains are information processing speed, alternating and divided attention, long-term memory, and executive functions, while word memory span and attention span are relatively preserved [4]. Finally, MS may cause psychological sequelae, such as malaise, fatigue, anxiety, depression, and low self-confidence, in addition to difficulties in sleeping and concentration [5]. All these symptoms lead MS to be one of the most burdensome neurological diseases [6].

As defined by the World Health Organization (WHO), disability arises out of the interaction between a health condition and the features of the environment that might facilitate or hinder a person's ability to carry out daily activities [7, 8]. Within this biopsychosocial framework, disability in MS tends to progress over time, thus limiting the individual functioning in daily-life activities [9], social participation [10], and quality of life [11]. Hence, disability assessment is essential to better understand the level of functioning and participation of people with health conditions and identify the necessary interventions [12].

In MS clinical practice, the Expanded Disability Status Scale (EDSS) [13] is considered the gold standard for the assessment of disability progression. Specifically, EDSS is an observer-rated scale used during neurological examinations, ranging from 0 (normal neurological status) to 10 (death due to MS). However, the EDSS has some methodological weaknesses, especially in reliability and sensitivity to change, but a sufficient validity criterion [14]. Furthermore, although the EDSS measures the impairments in all neurological functional domains (pyramidal, cerebellar, sensory, cognitive, brainstem, and sphincter), it weights higher the assessment of motor functioning, because, after a certain level of disability, the final score is strongly affected by the motor domain. Indeed, although the EDSS remains the gold standard for MS disability evaluation, it is not a comprehensive disability assessment tool as it focuses only on impairments and not on the impact of the environment. Coenen et al. [15] adopted the International Classification of Functioning, Disability and Health (ICF; [7, 16]) to evaluate the impact of MS on functioning, highlighting the impairments in cognition and restriction in social participation as key influencing factors of MS disability. The World Health Organization Disability Assessment Schedule 2.0 (WHODAS 2.0) is the tool for the assessment of functioning and disability, developed by the WHO according to the ICF biopsychosocial model [7, 17]. Specifically, the WHODAS 2.0 is a self-report questionnaire that investigates the subjects' level of functioning, thus allowing a more comprehensive assessment of disability by considering domains of activities and participations as well as the impact of the environmental factors on them. It could be considered a reliable disability assessment tool in pwMS [18], and higher levels of disability were self-reported at the WHODAS 2.0 by pwMS when compared with other chronic conditions [19].

In clinical practice, to slow or reduce the gradual worsening of MS symptoms and related disability, it is important to identify their influencing factors and allow early interventions on modifiable ones. In literature, several studies have already investigated them by assessing disability through the EDSS score and/or the conversion from relapsing–remitting (RRMS) to secondary progressive (SPMS) MS above all [13, 14]. Having in mind that EDSS is capturing impairments at body functions and body structures levels and not participation nor impact of environment, the presence of sphincter symptoms at onset (i.e., bladder or bowel symptoms) and early disease course outcomes (i.e., incomplete recovery after the first attack and short latency between first and second attacks) are associated with worse physical disability progression [20]. Furthermore, cognitive impairments (CI), especially in information processing speed and long-term memory, also play a key role in influencing disability progression [21, 22], including work disability [23]. When considering disability through other means, such as neuroimaging, a recent meta-analysis of magnetic resonance imaging studies revealed that brain imaging only moderately predicts disability progression [24].

So far, no studies have investigated which factors have an impact on MS disability measured by the WHODAS 2.0. However, we believe it could be useful in clinical practice to adopt the WHODAS 2.0, along with the EDSS, to measure the impact of the disease severity on a person's functioning and disability, thus allowing a more comprehensive understanding of the clinical profile of pwMS. Indeed, it could provide important suggestions on factors that might be modified to improve subjects' functioning, thus allowing intervention in disability progression trajectories. Furthermore, since MS usually occurs in young adults, often influencing their workability, identifying the barriers and the facilitators at personal and environmental levels will also have an impact on social as well as healthcare costs. This study is aimed at exploring the impact of clinical, psychological, cognitive, and social-support variables on disability, assessed with the WHODAS 2.0, in a sample of pwMS.

## 2. Method

### 2.1. Study Design and Recruitment of Participants

In this observational, cross-sectional, monocentric study, pwMS were enrolled voluntarily between 2015 and 2019, during hospitalization.

We included pwMS with age > 45 and a diagnosis of RRMS, SPMS, or primary progressive (PPMS) MS, according to the revised McDonald criteria [25]. Furthermore, participants were excluded if they presented other degenerative or autoimmune neurological diseases as well as major psychiatric disorders. The study was approved by the institute's ethical committee (Protocol No. 650/2014), and written consent was obtained from participants before enrolment.

### 2.2. The Research Protocol

#### 2.2.1. Outcome Measures

The short form of the WHODAS 2.0 [17] was administered to assess participants' subjective level of disability, within the framework of the biopsychosocial model. Specifically, the short form of the WHODAS 2.0 is composed of 12 items investigating difficulties in the level of functioning experienced during the previous 30 days in the following domains:

• Standing (i.e., “standing for long periods such as 30 min?”);
• Household responsibilities (e.g., managing finances and disciplining children);
• Learning new task (e.g., at home, work/school, and leisure activities);
• Joining in community activities (e.g., attending town meetings and sporting activities);
• Emotional affection (i.e., emotional impact due to health condition);
• Concentration (i.e., “concentrating on doing something for 10 min?”);
• Walking (i.e., “walking a long distance such as a kilometer [or equivalent]?”);
• Washing (i.e., “washing your whole body?”);
• Dressing (e.g., securing buttons and tying knots);
• Dealing with unknown people (e.g., interact with shopkeepers and ask someone for directions);
• Maintaining a friendship (e.g., interacting with friends in customary ways and participating in activities when invited);
• Work/school (e.g., attending on time and responding to supervision).

For each item, participant will be asked to answer from 1 (*none*) to 5 (*extreme*) and the summary score is then standardized from 0 (*no disabilit*y) to 100 (*full disability*). Since the WHODAS 2.0 total score is suitable for the use by pwMS [18], we adopted it as an index of overall disability.

#### 2.2.2. Sociodemographic Variables

Sociodemographic variables included sex, age, marital status (i.e., unmarried/celibate, married/cohabiting, separated/divorced, and widower), employment status (i.e., paid work, housewife, retired, unemployed, and unemployed for health reasons), and education (in years).

#### 2.2.3. Clinical, Neuropsychological, and Psychological Assessment

Participants underwent a neurological examination, including the EDSS [13], to assess objective physical disability as a proxy of MS severity.

Cognitive functioning was assessed through the Italian version of Rao's Brief Repeatable Battery of Neuropsychological Tests (BRB-NT), corrected for age, education, and sex [4]. Raw scores were transformed into corrected scores using published formulas [4]. The cognitive evaluations were done with the following tests, which assess the reported areas:

• Selective reminding test (SRT) assessed verbal learning and delayed recall. It contains a list of 12 words used in six consecutive learning trials and in one delayed trial, assessing the selective reminding test–long-term storage (SRT-LTS), the selective reminding test–consistent long-term retrieval (SRT-CLTR), and the selective reminding test–delayed recall (SRT-D). The participant is asked to repeat the 12 words read by the examiner and, at each subsequent trial, only the nonrepeated words are read again. All words correctly recalled in two consecutive trials participate in the long-term storage (LTS) score; all words consistently recalled on all subsequent trials are inserted in the consistent long-term retrieval (CLTR) results. The total sum of the items from both scores (LTS and CLTR) is used to gain raw SRT-LTS and SRT-CLTR scores. The raw score of the SRT-D is made by the total number of words recalled after the delayed period of 10–15 min.
• Symbol Digit Modalities Test (SDMT) assessed information processing speed. It presents nine symbols paired with corresponding 1–9 digits. Below, there is a random sequence of symbols, and the subject is asked to correctly fill in the corresponding digits as quickly as possible for a maximum time of 90 s.
• Paced Auditory Serial Addition Task (PASAT) assessed sustained attention and information processing speed. The participant hears a series of single-digit numbers presented once every 3 s in the first part (PASAT-3) or once every 2 s in the second part (PASAT-2). The subject has to sum pairs of numbers, adding each number to the previous. Sixty-one digits are presented for each part, and each part has a maximum of 60 correct answers.

For each subtest, cut-off scores were calculated at the fifth percentile of the corrected score. We considered CI when the scores in at least two tests are at least 1.5 standard deviation (SD) below the normative value [26].

Finally, participant-reported outcome measures were adopted to collect psychological and social-support variables. Specifically, the State-Trait Anxiety Inventory-Form Y (STAI-Y) [27] is composed of 40 items divided into two subscales that evaluate the state (i.e., how anxious the person is at the time of the questionnaire) and trait (i.e., how anxious the person is in general) anxiety. Each item is rated on a 4-point Likert scale (1–4), with higher scores indicating higher anxiety levels; the total score ranges from 20 to 80. Symptoms of depression were measured through the 21-item Beck Depression Inventory (BDI)-II [28], which assesses cognitive (i.e., pessimism, guilt, and low self-esteem) and somatic–affective features of depressive symptoms (i.e., crying, loss of interest and energy, and agitation). The BDI-cognitive score range is 0–24, the BDI-somatic–affective score range is 0–39, and the total BDI-II score range is 0–63. Higher scores reflect higher depressive mood: A BDI-II total score range of 14–19 is indicative of mild depressive symptoms, a range of 20–28 of moderate depressive symptoms, and ≥ 29 of severe depressive symptoms.

The Medical Outcome Study–Social Support Survey (MOS-SSS) [29] was administered to evaluate the level of subjects' perceived social support. It is composed of 19 items investigating emotional support and affective, tangible, and positive social interactions. Each item requires an answer on a 5-point Likert scale (1–5), with higher scores representing better support. The total score is derived from the sum of each item score.

### 2.3. Data Analysis

Data were analyzed with Statistical Package for Social Sciences (IBM SPSS Statistic, version 24).

For descriptive analysis, we used frequencies and percentages for categorical variables, while mean and SD were used for continuous ones. For the neuropsychological tests administered, corrected scores were reported, according to the Italian normative data [4]. A multivariable regression analysis with a forward procedure was applied to target the WHODAS 2.0 as an outcome and all the clinical, neuropsychological, and psychosocial variables as predictors. The alpha level was set up at *p* < 0.05 as a threshold for variable retention so that only independent significant predictors were included in the final model.

*F*-statistic was used to address the difference between regression and residual mean square. The variance explained by the forward regression procedure was measured with *R*^2^ change, putting significance at *p* < 0.05 level. Multicollinearity was evaluated using variance inflation factor (VIF) and tolerance tests: VIF < 5 and tolerance > 0.20 indicated the absence of multicollinearity; variables with a correlation coefficient > 0.70 were excluded for the risk of multicollinearity. Thus, we selected the variables with the higher predictive power as shown by beta coefficients. Homoscedasticity was addressed by drawing a plot with regression's standardized residuals on the *Y*-axis and standardized predicted values on the *X*-axis: A pattern with dots randomly and evenly dispersed indicates that homoscedasticity was not violated.

## 3. Results

Data from 151 pwMS were analyzed. The sample was mainly composed of female participants (*N* = 93; 61.6%) with a mean age of 51.6 (SD = 5.83). Most subjects have RRMS (*N* = 130; 86.0%) with a mean disease duration of 11.96 (SD = 8.75) years.  Table 1 summarizes the general sample information.

In the final regression model, State-Trait Anxiety Inventory-Form S (STAI-S), BDI-II, and EDSS resulted as independent predictors and the model accounted for 70.5% of WHODAS 2.0 variability (Adj.*R*^2^ = 0.705; *p* < 0.001; see Table 2).

## 4. Discussion

We found that symptoms of anxiety and depression and EDSS score have an impact on perceived disability in pwMS, thus accounting for the 70% of WHODAS 2.0 variation. When we analyzed the final models in depth, considering the role played by each variable, some main results emerged.

The first finding showed that disease severity, measured through the EDSS, thus focusing on impairments of mobility, has the most predictive power on the disability level, accounting for ~50% in the initial step of the multivariable model and resulting in the variable most associated with the WHODAS 2.0 total score in the final model. This result is consistent with a previous finding [30] reporting that mild neurological deficits (i.e., better motor abilities of both upper and lower extremities than subjects with moderate neurological deficits), measured through the EDSS, were associated with a higher level of functioning. Meanwhile, lower WHODAS 2.0 scores, indicating the dimensions of lower disease severity, predicted the subjects' functioning in different domains of life. A previous study has shown that the transition of the EDSS score from 1.0–3.0 to 3.5–5.5 significantly affects all aspects of functioning [9] and, therefore, pwMS requires support in the evolution of the disease. However, the relevant finding of our research is that the final variance explained by the forward regression analysis accounted for around 70% when the psychological variables were considered: State anxiety and depressive mood, measured through the STAI-S and the BDI-II, respectively, accounted for an additional 20% of the variance explained by the model, particularly given predictive power importance to the BDI-II scores (*R*^2^ change: 0.191). This association among psychological variables and subjective disability levels is not surprising, since the psychological symptoms may be ascribed to many of all the facets related to MS symptomatology affecting the overall functioning of patients, reducing physical functions, raising difficulties in carrying out the activities of daily living, and reducing independence. Furthermore, these symptoms might play a very relevant role in relevant life areas causing early school dropout, problems with employment, sexual difficulties, and issues with establishing relationships with family members and friends [31]. Butler, Matcham, and Chald [32], for example, found that higher levels of anxiety were associated with lower levels of perceived social support in pwMS; this result may be related to the fact that some people with anxiety may have distorted perceptions of the availability of social support [32].

Regarding depressive symptoms, previous findings demonstrated that the somatic–affective component strongly affected the level of disability [33]. This result seems to be in line with this study: Our subjects enrolled showed higher means of somatic–affective symptoms than cognitive ones (means somatic–affective BDI-II: 7.71 ± 5.74 SD vs. means cognitive BDI-II: 3.11 ± 3.87 SD) and this even when only the overall score of BDI-II was entered in the model.

Arnett, Higginson, and Randolph [34] have found a correlation between increased depressive symptoms, anxiety, irritability, anger, and somatic disturbances over time and a decreased use of active coping strategies, leading to a higher level of MS overall disability. Finally, the frequency of depression in MS addresses the risk of suicide and suicidal attempts, particularly in younger males, within the first 5 years of diagnosis. In this way, Feinstein found a lifetime prevalence for suicidal intent of 28.6% and that 6.4% of subjects had already attempted suicide; moreover, living alone, severe depression, and alcoholism were risk factors for suicidal intent [35]. In summary, although the WHODAS 2.0 was not designed to measure depression or anxiety per se [36], it is sensitive to psychological symptoms [36, 37]. However, the causal relationship between the aforementioned constructs is not clear [38] because psychological sequelae may result from the patient's disability condition but, on the other hand, higher levels of anxiety and depression could worsen the perception of subjective disability.

Second, available literature about individuals with chronic illness showed that those who have consistent social support usually performed better. Social support is important in several different forms, specifically emotional and logistical support. For example, Mohr et al. [39] found that pwMS commonly experience deteriorated relationships and have feelings of victimization after being diagnosed. In our study, social support was measured using the MOS-SSS [29], which evaluates the subjective level of perceived social support, but it did not show a significant association with the WHODAS 2.0 total score. This result is in contradiction with several previous studies addressing social support and disability in pwMS and the general population [40–42]. For example, Leonardi et al. [41] showed that environment and social networks are core determinants of health and disability in an aging population as well as Guastafierro et al. [40] showed that higher scores in the social network index, as measured through the social network index and social support, as measured through the OSLO-3 Social Support Scale [43], predicted an increase in the quality of life and the reduction of disability, respectively, in a sample of older adults in Italy. Finally, Raggi et al. [42] found that difficulties with interpersonal relationships negatively affect MS patients' ability to work, so a negative work environment, defined as a nonsupportive workplace and workplace inaccessibility, is associated with higher reduction of the proportion of worked hours and increase of the likelihood of having left work. Thus, specific tools for the evaluation of social support seem to have a general impact on the overall sense of positive well-being and health-promoting behaviours in the general population and pwMS [44].

The last result showed that the cognitive functioning of pwMS was not significantly associated with the disability level. This result might be due to the low prevalence of CI of our sample (28.6% had > 2 impaired tests at the Rao's Brief Repeatable Battery (BRB); see [26]) and that our participants were mainly middle-aged with high levels of formal education. Although cognitive tests were corrected for age, education, and sex [4], a different and more inclusive approach to the definition of CI for our MS sample, based on a categorical definition of cognitive dysfunction (i.e., a score below 2 SD from the mean [45]), could have led to a more stringent definition of CI.

Although the relationship between clinical, psychological, cognitive, and social-support variables with disability levels is widely investigated in MS literature [46–48], also uncovering their impact on quality of life [49, 50], this is the first study that explored these associations taking into account the WHODAS 2.0. Indeed, disability levels in pwMS are usually assessed through the EDSS, thus encompassing only the level of disease severity related to body functions and structures. Instead, by adopting a biopsychosocial perspective, that enables considering subjects' self-reported evaluation of functioning in the actual contexts of life, this study confirmed the existence of an association between clinical and psychological variables with disability level by considering multiple levels and profiles of disability.

Therefore, although the EDSS remains the gold standard of MS clinical evaluation, it is not a comprehensive disability assessment tool to assess the MS's impact on functioning, including cognition, participation, and environment. Thus, adding a more complete evaluation of anxiety and depressive symptoms could be useful in clinical practice to plan tailored and personalized psychological interventions (i.e., cognitive behavioural therapy and acceptance or commitment therapy) to reduce the disability levels of pwMS.

Some limitations should be acknowledged in our study. First, the cross-sectional design of the study did not allow us to draw a firm conclusion about the relations between variables: Therefore, caution is needed in the interpretation of our results, which also requires that the role of possible biases, such as that played by psychological factors, be considered. Second, subjects were enrolled on a single third level of the care center and had a relatively low EDSS score. Furthermore, most participants had an RRMS phenotype: Therefore, caution is needed in generalizing results to a wider and more heterogeneous population of pwMS. Finally, comorbidities or CI might have occurred also independently from the MS disease: We relied on a set of cognitive tests measuring relevant cognitive functions (i.e., verbal learning, long-term memory, attention, and processing speed), but it can be argued that a more comprehensive testing could have brought more information.

## 5. Conclusion

The results of this study suggest that together, symptoms of anxiety, depression, and MS severity measured by EDSS accounted for 70% of pwMS overall disability, as scored by the WHODAS 2.0. Our results are of interest to clinicians as they provide insights on the need to widen the approach to reduce disability in pwMS, thus including as possible all modifiable factors at a personal level (i.e., physical and psychological factors), as well as at an environmental level (i.e., evaluating main life domains and person social network). Identifying all elements in a biopsychosocial perspective allows to identify the right and prompt intervention in a more tailored manner for each pwMS.

Our research highlights that not only physical impairments need to be considered but also psychological symptoms, which are promising targets for psychosocial interventions to reduce the burden of illness and enhance the recovery process of pwMS. Therefore, our research provides a valuable indication to add careful monitoring also of anxiety, depressive symptoms, social networks, and impact on major life areas on top of the careful evaluation of levels of physical disability and thus to operationalize in a biopsychosocial perspective the approach to reduce pwMS disability.

## Figures and Tables

**Table 1:** Characteristics of enrolled pwMS.

	**Results as mean (SD) or ** **N** ** (%)**
*Sociodemographic variables*	
Age, in year	51.6.±5.83
Sex	
Female	93 (61.6%)
Male	58 (38.4%)
Marital status	
Unmarried/celibate	24 (15.9%)
Married/cohabiting	112 (74.2%)
Separated/divorced	13 (8.6%)
Widower	2 (1.3%)
Education, in year	12.84 ± 3.7
Employment status	
Paid work	101 (66.9%)
Housewife	10 (6.6%)
Retired	18 (11.9%)
Unemployed	11 (7.3%)
Unemployed for health reasons	11 (7.3%)
*Clinical variables*	
EDSS, median (IQR)	2.0 ± 1.5
MS phenotype: RR	130 (86.1%)
Disease duration, in years (range)	11.96 ± 8.75 (0–37)
*Neuropsychological variables*	
Cognitive testing (corrected scores)	
SRT-LTS	34.20 ± 13.9
SRT-CLTR	25.09 ± 14.25
SRT-D	6.42 ± 2.75
SDMT	41.05 ± 11.87
PASAT-3	33.98 ± 14.26
Cognitive dysfunction (≥ 2 impaired tests at the BRB)	19 (28.6%)
*Psychological variables*	
STAI	
STAI-State	50.24 ± 10.8
STAI-Trait	50.66 ± 10.1
Relevant anxiety symptoms	16.5 (25%)
BDI-II	
BDI-cognitive	3.11 ± 3.87
BDI-somatic–affective	7.71 ± 5.74
BDI-total	10.8 ± 8.87
Relevant depressive symptoms	11.6 (17.5%)
MOS-SSS total	3.90 ± 1.0
*Outcome measure*	
WHODAS 2.0 (summary score)	25.1 ± 19.86

Abbreviations: BDI-II, Beck Depression Inventory; BRB, Rao's Brief Repeatable Battery; EDSS, Expanded Disability Status Scale; MOS-SSS, Medical Outcome Study–Social Support Survey; PASAT-3, Paced Auditory Serial Addition Task; pwMS, persons with multiple sclerosis; RR, relapsing–remitting; SDMT, Symbol Digit Modalities Test; SRT-CLTR, selective reminding test–consistent long-term retrieval; SRT-D, selective reminding test–delayed recall; SRT-LTS, selective reminding test–long-term storage; STAI, State-Trait Anxiety Inventory; WHODAS 2.0: World Health Organization Disability Assessment Schedule.

**Table 2:** Multivariable linear regression model with a forward procedure predicting variation of the World Health Organization Disability Assessment Schedule (WHODAS 2.0) in pwMS.

**Study variables**	**Multivariable linear regression**					
	**Initial model**		**Second model**		**Final model**	
	**β**	**t** ** (** **P** **)**	**β**	**t** ** (** **P** **)**	**β**	**t** ** (** **P** **)**
Constant		1.073 (0.285)		−2.810 (0.006)		−3.609 (< 0.001)
EDSS	8.675	12.339 (< 0.001)	7.429	13.096 (< 0.001)	7.190	12.769 (< 0.001)
BDI-II	—	—	1.005	9.663 (< 0.001)	0.835	6.944 (< 0.001)
STAI-S	—	—	—	—	0.265	6.944 (0.009)

*R* ^2^	0.505		0.697		0.711	
Adj.*R*^2^	0.502		0.693		0.705	
*R* ^2^ change	0.505		0.191		0.014	
*F* (*p*)	152 (< 0.001)		170 (< 0.001)		120 (< 0.001)	

Abbreviations: BDI-II, Beck Depression Inventory-II; EDSS, Expanded Disability Status Scale; pwMS, persons with multiple sclerosis; STAI, State-Trait Anxiety Inventory.

## Data Availability

Data will be available on request.
